# Specific genomic aberrations in primary colorectal cancer are associated with liver metastases

**DOI:** 10.1186/1471-2407-10-662

**Published:** 2010-12-02

**Authors:** Sjoerd C Bruin, Christiaan Klijn, Gerrit-Jan Liefers, Linde M Braaf, Simon A Joosse, Eric H van Beers, Victor J Verwaal, Hans Morreau, Lodewyk F Wessels, Marie-Louise F van Velthuysen, Rob AEM Tollenaar, Laura J van't Veer

**Affiliations:** 1 Division of Experimental Therapy, Department of Surgery, The Netherlands Cancer Institute - Antoni van Leeuwenhoek Hospital, Plesmanlaan 121, 1066CX Amsterdam, The Netherlands; 2 Department of Molecular Biology, The Netherlands Cancer Institute - Antoni van Leeuwenhoek Hospital, Plesmanlaan 121, 1066CX Amsterdam, The Netherlands; 3 Delft University of Technology, Delft Bioinformatics Lab., PO Box 5031. 2600 GA, Delft, The Netherlands; 4 Department of Surgery, Leiden University Medical Center, Albinusdreef 2, 2333 ZA, Leiden, The Netherlands; 5 Division of Experimental Therapy, The Netherlands Cancer Institute - Antoni van Leeuwenhoek Hospital, Plesmanlaan 121, 1066CX Amsterdam, The Netherlands; 6 Institute of Tumor Biology, Center of Experimental Medicine, University Medical Center Hamburg-Eppendorf, Martinistrasse 52, 20246 Hamburg, Germany; 7 Department of Surgery, The Netherlands Cancer Institute - Antoni van Leeuwenhoek Hospital, Plesmanlaan 121, 1066CX Amsterdam, The Netherlands; 8 Department of Pathology, Leiden University Medical Center, Leiden, The Netherlands; 9 Department of Pathology, The Netherlands Cancer Institute - Antoni van Leeuwenhoek, Plesmanlaan 121, 1066CX Amsterdam, The Netherlands; 10 Division of Experimental Therapy, Department of Pathology, The Netherlands Cancer Institute - Antoni van Leeuwenhoek Hospital, Plesmanlaan 121, 1066CX Amsterdam, The Netherlands

## Abstract

**Background:**

Accurate staging of colorectal cancer (CRC) with clinicopathological parameters is important for predicting prognosis and guiding treatment but provides no information about organ site of metastases. Patterns of genomic aberrations in primary colorectal tumors may reveal a chromosomal signature for organ specific metastases.

**Methods:**

Array Comparative Genomic Hybridization (aCGH) was employed to asses DNA copy number changes in primary colorectal tumors of three distinctive patient groups. This included formalin-fixed, paraffin-embedded tissue of patients who developed liver metastases (LM; n = 36), metastases (PM; n = 37) and a group that remained metastases-free (M0; n = 25).

A novel statistical method for identifying recurrent copy number changes, KC-SMART, was used to find specific locations of genomic aberrations specific for various groups. We created a classifier for organ specific metastases based on the aCGH data using Prediction Analysis for Microarrays (PAM).

**Results:**

Specifically in the tumors of primary CRC patients who subsequently developed liver metastasis, KC-SMART analysis identified genomic aberrations on chromosome 20q. LM-PAM, a shrunken centroids classifier for liver metastases occurrence, was able to distinguish the LM group from the other groups (M0&PM) with 80% accuracy (78% sensitivity and 86% specificity). The classification is predominantly based on chromosome 20q aberrations.

**Conclusion:**

Liver specific CRC metastases may be predicted with a high accuracy based on specific genomic aberrations in the primary CRC tumor. The ability to predict the site of metastases is important for improvement of personalized patient management.

## Background

Colorectal cancer (CRC) is the second leading cause of cancer death in the Western world. Every year one million people worldwide will develop CRC[1]. The overall five-year survival is 57% and up to 50% of all patients will eventually develop metastases. Metastases are responsible for the great majority of cancer deaths, mainly metastatic liver disease. Even with surgery and modern chemotherapy most metastases are eventually fatal.

Besides lymphatic and haematogenous dissemination, CRC can spread into the intra-abdominal cavity and cause peritoneal metastases (PM).

Of all patients who die of advanced colorectal cancer, 60-70% show evidence of liver metastasis. Even with the use of targeted drugs, the overall survival in patients with non-resectable CRC liver metastases is only 2-years. In patients with resectable liver metastasis a 5-year survival of 30% can be achieved and up to 20% of the population will still be alive after 10 years. Eligibility for hepatic surgery depends on the possibility that all metastases are resectable and adequate liver preservation can be maintained. Therefore early detection of liver metastases is of utmost priority and will result in more radical surgery and thus long term survival[2].

Similar to liver metastasis, peritoneal metastasis is uniformly seen as a fatal condition. However, in the last decade survival has improved due to aggressive cytoreductive surgery in combination with hyperthermic intraperitoneal chemotherapy (HIPEC). Several predictors of outcome after HIPEC treatment, such as completeness of cytoreduction and number of affected intra-abdominal tumor regions, have been described. Patients who underwent complete cytoreduction in combination with HIPEC showed a 5-year survival of 22-49%. Early detection of peritoneal metastases will result in a better cytoreduction and less affected intra-abdominal regions[3]. In conclusion, early detection of liver and peritoneal metastasis will result in a tailored follow-up program and through this better patient outcome.

Accurate staging of CRC with clinicopathological parameters is important in predicting prognosis and guiding treatment but can currently not predict the site of metastases. Therefore, understanding of the molecular and cellular mechanisms underlying colorectal cancer formation, in particular progression to site of metastases is of utmost importance.

The development of CRC arises and develops through the adenoma-carcinoma sequence. This adenoma-carcinoma sequence is a well defined pathway of histopathological stages, each characterized by distinct mutations in oncogenes and tumor suppressor genes [4,5]. Two molecular pathways have been well described; the microsatellite instability and the chromosomal instability pathway (reviewed by Jass et al) [4,6,7]. The majority of CRC (85%) are chromosomally unstable [5], characterized by allelic losses, chromosomal amplifications and translocations [8], whereas mismatch repair deficiency is the underlying cause of the remainder of CRCs.

Cardoso et al. reviewed multiple studies that have reported on the existence of chromosomal abnormalities and gene expression profiles in CRC [8]. With this approach they described several specific chromosomal loci and corresponding genes which play an important role in colorectal cancer progression. A meta-analysis by Diep et al. on 31 comparative genomic hybridization (CGH) studies, comprising a total 859 CRCs, described chromosomal alterations that occurred early in the establishment of primary CRC, as well as those that are present in the different Dukes' stages and in liver metastases[9].

So far, a number of chromosomal aberrations have been related to liver metastases in CRC [9-13]. Metha et al. studied the relation between the extent of chromosomal instability and the survival of patients with liver metastases. They showed that with a larger chromosomally unstable fraction in the liver metastases, survival for patients was better[14].

Various studies have described prognostic gene expression profiles for CRC patients although these profiles have very few genes in common [15-20]. Gene expression profiles have been described for breast cancer that predict site specific recurrence e.g., bone and lung metastases[21-23]. However, gene expression or genomic profiles in CRC that predict site specific recurrence have not been well studied.

Here, we investigated genome-wide chromosomal aberrations in defined groups of primary colorectal tumors to determine copy number signatures for site specific metastases. The understanding of the molecular and cellular mechanisms underlying colorectal cancer formation, progression to malignancy and site specific metastases are important to perform targeted follow-up and eventually develop targeted therapy in patients with CRC.

## Methods

### Patients and Tumor Specimens

We have used formalin-fixed, paraffin embedded (FFPE) primary colorectal tumors of three different groups. 356 patients were selected on the basis of their disease outcome in follow-up. Patients, who had developed metastases were treated in two specialized centers: liver metastasis (LUMC), peritoneal metastasis (NKI-AVL), whereas all of the non-metastasis group were treated for their primary tumor at the NKI-AVL. For the metastatic groups primary tumor material was obtained from several different pathology laboratories in the Netherlands. In total, 119 samples were retrieved, of which 98 samples remained after quality control. One group (n = 36) were patients who have been treated with Isolated Hepatic Perfusion (IHP) for the treatment of CRC metastases confined to the liver (LM)[24]. The second group (n = 37) were patients who have been treated with hyperthermal intraperitoneal chemotherapy (HIPEC) for the treatment of CRC metastases confined to the peritoneum (PM) [25]. The third group (n = 25) consisted of patients with CRC who did not develop metastases (M0). In this M0 group only two patients had a minimal follow-up of 12 months. All the others had a follow-up of at least 36 months. That implicates that 92% had a follow-up of at least 3-years. The M0 group showed a median follow-up of 103 months. The LM and PM groups were carefully screened to ensure these were free of distant metastases at time of treatment, other than the liver or the peritoneal surface, respectively [25,26]. For that reason we characterized these two groups as single organ specific CRC metastases. In this study none of the primary tumors were treated neo-adjuvant with chemotherapy. We have used anonymized patient tissue material of patients who were consented for a HIPEC procedure (PM) or Liver perfusion (LM), or had not opted out for making left-over tissue available for research (M0), which followed standard procedures operational in the respective hospitals approved by the institutional IRB's or Board, which ever one was appropriate. Tissue handling and anonymization followed the Helsinki declaration.

### Comparative Genomic Hybridization

DNA copy number changes were investigated using the 3.5 k bacterial artificial chromosomes (BAC) array performing array comparative genomic hybridization (aCGH)[27]. The human 3600 BAC/PAC genomic clone set, covering the full genome at 1 Mb spacing used for the production of our arrays, was obtained from the Welcome Trust Sanger Institute http://www.sanger.ac.uk/. Information on this clone set can be obtained at the BAC/PAC Resources Center Web Site http://bacpac.chori.org. The whole library was spotted in triplicate on every slide. To prevent slide batch spotting bias, samples were hybridized in random order http://microarrays.nki.nl/.

DNA isolation was performed as described earlier[28]. Briefly, genomic DNA was isolated by proteinase K digestion after deparaffination from 10 × 10 μm FFPE tissue sections containing at least 70% tumor cells from both the M0 and PM group. For the LM group DNA was isolated from FFPE tissue block punches. These punches were taken out of the tissue blocks in the area with at least 60% tumor cells. Reference DNA was isolated from peripheral blood lymphocytes from six healthy male individuals. It was pooled and sonicated until its median fragment length was similar to that of the test samples.

DNA quality was tested by measuring the maximum possible length of DNA to be amplified by a multiplex PCR as described elsewhere [29]. This mulitplex PCR produces DNA fragments of 100, 200, 300, and 400 bp. Samples of which at least 200 base pair fragments could be produced were of sufficient quality to be analyzed with aCGH[29] We initially selected 119 cases of which 98 passed the quality control. There were no significant differences in tumor/patient characteristics in the selected versus initial group.

Tumor DNA labelling was performed with ULS-Cy3 and ULS-Cy5 conjugates from the Universal Linkage System (ULS Kreatech Biotechnology, Amsterdam the Netherlands) [30]. Hybridisations on the arrays were done at our Central Microarray Facility, as described previously[27]. Data processing of the scanned microarray slide included signal intensity measurement with the ImaGene software program, followed by median pin-tip (*c.q*. sub array) normalization. Intensity ratios (Cy5/Cy3) were log_2_-transformed and triplicate spot measurements were averaged[27]. Microarray data have been deposited in NCBI's Gene Expression Omnibus (Edgar et al Nucleic Acids Res 2002) and are accessible through GEO Series accession numbers GSE20496.

For MSI analyses, DNA from paired normal and tumor samples was evaluated after PCR and fragments were analyzed using 2 mononucleotide markers (BAT 25 and BAT 40), and 6 dinucleotide markers (D1S158, D2S123, D5S346, D9S63, D17S250 and D18S58). A tumor was considered to be MSI-high in case three or more markers showed instability, MSI-low when only one marker showed instability and MSI-stable when none of the markers showed instability[16].

### Data analysis

**Figure 1:**
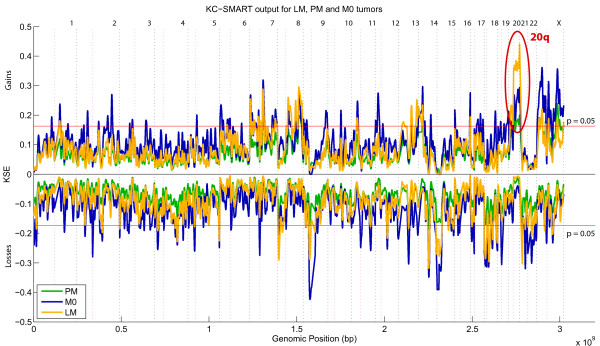
**Visualisation of a three group KC-SMART analysis**. This figure shows the Kernel Smoothed Estimated (KSEs) for the three groups analyzed in this study (Liver Metastases (LM), Peritoneal Metastases (PM) and No metastases (M0)). All chromosomes are plotted head-to-tail, Black dotted lines denote the end of chromosomes and magenta dotted line denote centromere locations.

KC-SMART, a recently developed algorithm for identifying recurrent copy number changes, was used to identify specific locations of genomic gains and losses recurring at significant levels within the various groups [31]. Briefly, an aggregate profile of the aberrations is created by adding either the positive log_2 _values or the negative log_2 _values across all tumors. These aggregate profiles (one for gains and one for losses) are smoothed using locally weighted Gaussian kernel convolution, resulting in the Kernel Smoothed Estimate (KSE). KSEs computed for many randomized instances of the dataset are employed to generate a null distribution and establish a (multiple testing corrected) significance threshold (p = 0.05, Figure 1). All aberrations in the smoothed profile computed on the real data that exceed this threshold are deemed to be significantly recurrent in the dataset.

In order to detect consistent differences in copy number changes between two groups, we employed comparative KC-SMART. This approach employs the signal-to-noise-ratio (SNR) to quantify the differences between the profiles of two groups of tumors. The SNR is a ratio of the absolute difference of the means of the classes and the sum of the average within-class standard deviation, analogous to the t-statistic. In order to calculate the SNR we produce smoothed profiles as described above for single tumors, instead of on an aggregate of the entire group. This smoothed profile is constructed on a grid with a grid spacing of 50,000 base pairs. For each grid point we calculate the SNR between two groups. To establish significance we permute the group labels 1000 times. Based on the results of this permutation, we are able to calculate the false discovery rate (FDR), and define a 1% FDR threshold. Any region for which the SNR exceeds this threshold, is significantly differentially aberrant between the two groups.

Logistic regression and its confidence intervals were calculated using the glm and stats packages of the statistical analysis software R http://www.r-project.org. Approximate absolute copy numbers for a certain genomic region were calculated from the averaged log_2 _measurements over all BAC clones mapped to the region.

We created two classifiers for organ specific metastases, using a shrunken centroids classifier (Prediction Analysis for Microarrays: PAM http://www-stat.stanford.edu/~tibs/PAM) on the aCGH data. The first classifier was trained to distinguish the LM group from a group consisting of both the PM and M0 groups together. The second classifier was trained to distinguish the PM group from the M0 group (additional figures).

## Results

### Patient and tumor characteristics

Patient and tumor characteristics like gender, tumor differentiation and MSI status, were not significantly different between the three patient groups (M0, PM and LM respectively) (Table 1). Groups differed for some characteristics that could be partly accounted for by the different selection criteria employed for each of the groups. For instance, for the tumor location more right-sided tumors were included in the M0 group, whereas the PM and LM group consisted of more left-sided colon and rectum tumors. There were relative more T4 tumors in the PM group compared with the M0 and LM group. Naturally, the M0 group showed no stage 4 patients and was therefore significantly different from the LM and PM group. The LM group had the highest percentage of Stage 4 patients.

**Table 1:** Patient and tumor characteristics No metastases (M0), Peritoneal Metastases (PM) and Liver Metastases (LM) patients

		M0			PM			LM			p-value**
		**25**	**Percentage**		**37**	**Percentage**		**36**	**Percentage**		

**Gender**	Male	14	56%		21	57%		27	75%		
											
	Female	11	44%		16	43%		9	25%		0,185

**Mean age at diagnosis**				64,21			53,23			54,66	

**Location primary tumor**	Right colon	11	44%		11	30%		4	11%		
											
	Left colon	9	36%		22	59%		22	61%		
											
	Rectum	5	20%		4	11%		10	28%		0,027

											

**T-status**	T1	0	0%		0	0%		0	0%		
											
	T2	0	0%		1	3%		4	11%		
											
	T3	21	84%		25	67%		30	84%		
											
	T4	4	16%		11	30%		1	3%		0,01
											
	unknown	0	0%		0	0%		1	3%		

											

**N-status**	N0	19	76%		17	47%		13	36%		
											
	N+	6	24%		19	53%		22	61%		0,01
											
	unknown	0	0%		0	0%		1	3%		

											

**Stage**	1	0	0%		1	3%		0	0%		
											
	2	16	64%		5	13%		3	8%		
											
	3	9	36%		13	35%		7	20%		
											
	4	0	0%		18	49%		26	72%		0

**Presentation**											0,009

	Metachrone	25	100%		19	51%		8	22%		
										
	Synchrone				18	49%		26	72%		
											
	Unknown				0	0%		2	6%		1,00E-06

											

**Differentiation**	Good	0	0%		3	8%		3	12%		
											
	Moderate	21	88%		27	73%		17	65%		
											
	Poor	3	13%		5	14%		6	23%		
											
	Other type	0	0%		2	5%		0	0%		0,3

**Overall survival**											
	
**Median in months (range)**				103 (12-279)			22 (2-120)			26 (0-108)	

											

**MSI status**	Stable	21	91%		33	97%		30	100%		
											
	Low	2	9%		0	0%		0	0%		
											
	High	0	0%		1	3%		0	0%		0,124

											

**Adjuvant treatment**	No	16	64%		25	68%		34	94%		
											
	Chemo	6	24%		12	32%		2	6%		
											
	Unknown	3	12%		0	0%		0	0%		0,014

** = Pearson Chi-Square Test

MSI status = Micro Satellite Instability status

Almost all patients were MSI stable (including MSI low) except for 1 patient in the PM group.

### Comparative Genomic Hybridization and KC-SMART analysis

Genomic DNA of 98 tumor samples was hybridized to BAC-clone microarrays to obtain, for each BAC clone on the array, a log^2 ^ratio of the fluorescence intensity ratio of the sample versus the reference for each of the three groups. We first analyzed the three groups (LM, PM, M0) separately with KC-SMART to detect regions of common aberrations across the samples in a group. See Figure 1.

The KC-SMART analysis showed that the three groups had an overall similar pattern of chromosomal aberrations, with the Pearson's correlation coefficient between the KSEs of the groups being 0.84 for the gains and 0.90 for the losses (Figure 1). Although the genomic instability in the three groups was similar overall, chromosome 20 was an exception.

**Figure 2:**
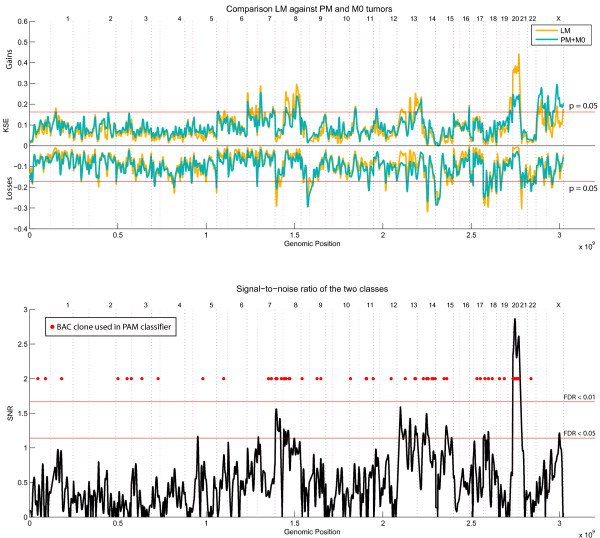
**Gains and losses of the Liver Metastases (LM) samples versus the Peritoneal Metastases (PM) and No metastases (M0) samples**. Shown here is the KSE (Kernel Smoothed Estimated) of the LM and PM groups in the upper panel. The lower panel shows the signal-to-noise (SNR) calculated between the two groups. The red dots represent the BAC clones used for the LM-PAM classifier. All chromosomes are plotted head-to-tail, Black dotted lines denote the end of chromosomes and magenta dotted line denote centromere locations. FDR = False Discovery Rate.

**Figure 3:**
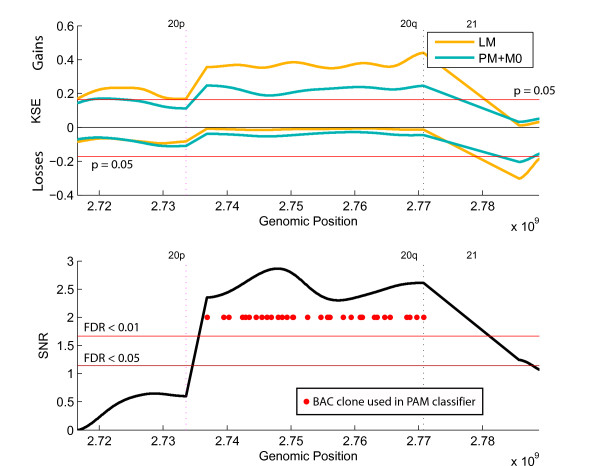
**Gains and losses of the Liver Metastases (LM) samples versus the Peritoneal Metastases (PM) and No metastases (M0) samples**. Panel showing a zoom of chromosome 20.

To better identify chromosomal aberrations specific for the liver metastases group, we compared this group with the other two groups (M0 and PM) combined (Figure 2 and 3). Chromosome 20q was significantly more gained in the LM group compared with the other samples (Kernel Smoothed Estimate (KSE); p < 0.05), Bonferroni multiple testing corrected, Figure 2 top panel, aberrations exceeding the red lines). The comparative KC-SMART analysis yielded chromosome 20q with a FDR < 0,01 and several other regions with a FDR of <0.05 (Figure 2; lower panel). These other significant gains (FDR < 0.05) and losses specific for the LM group were located on chromosomes 5q, 7q, 8p, 8q,13q, 14q, 15q, 18p, 18q (Figure 2; lower panel and additional file 1 figure S1). Further investigation of the region gained on chromosome 20 showed that BAC clones encompassing the whole q-arm are gained in the LM group (Figure 3; KSE and SNR upper and lower panel respectively).

Logistic regression analysis to predict liver metastases recurrence based on the mean 20q profile of gains/losses for each group separately, showed for every 0.1 log^2 ^20q amplification an odds ratio of 16.2 (90% CI: 2.3-141.2) for developing liver metastases. To test whether nodal status and the 20q mean log^2 ^value are independent predictors of liver metastases, we added both terms to a logistic regression and determined the significance of the corresponding regression coefficients. Both coefficients were significant (20q: P < 0.00003, nodal status: P < 0.024). This shows that our prediction of liver metastases based on mean 20q log^2 ^value is independent of nodal status.

**Figure 4:**
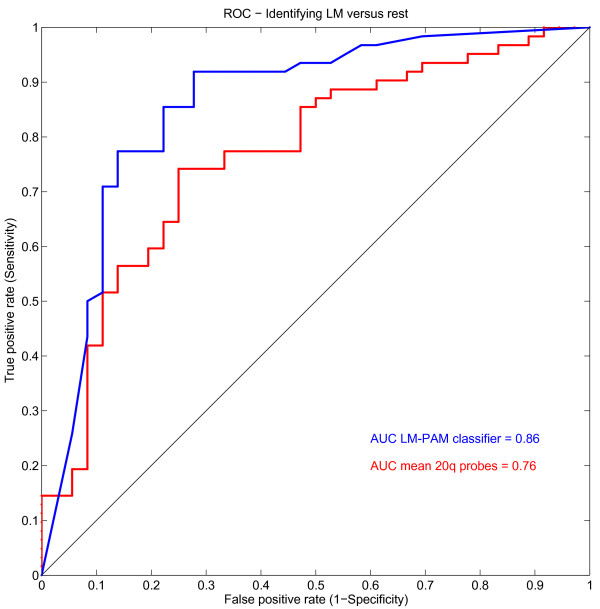
**ROC curve when using 20q as a predictive value for liver metastasis**. This figures shows the Reciever-Operating Characteristic (ROC) curve for using the mean log^2 ^ratio of the 20q BAC clones (red) or the mean log^2 ^of the BAC clones that resulted from the LM-PAM classfier (blue). AUC = Area Under the Curve.

To investigate the predictive association of the 20q amplification we constructed a Receiver-Operator-Characteristic curve (ROC-curve) for the average log^2 ^values of the chromosome 20q BAC clones. This simple measure already results in an Area Under the Curve (AUC) of 0.76 for predicting LM (Figure 4 red line).

**Figure 5:**
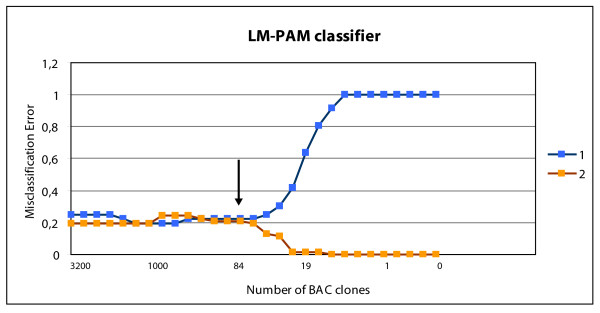
**PAM analysis of the Liver Metastases (1) versus the rest of the group (Peritoneal Metastases & No metastases) (2)**. The arrow indicates the cutoff location of the LM-PAM classifier and number (84) of BAC clones in classifier. PAM = Prediction Analysis for Microarrays. LM--PAM Classifier = Liver Metastases- Prediction Analysis for Microarrays classifier.

A LM-PAM classifier was able to separate the LM group from the other groups (M0 and PM) with 80% accuracy ((Figure 5 misclassification error 20%) (78% sensitivity and 86% specificity)). This LM-PAM classifier included 84 BAC clones and was significantly enriched for probes located on chromosome 20q (Additional file 2 table S1; marked green). Using the per-tumor class probabilities derived from the PAM classifier we also built a ROC-curve for this classifier which resulted in an AUC of 0.86 (Figure 4 blue line). Chromosome 20 is represented by 65 BAC clones on the BAC-array; 21 of these clones represent the p-arm and 44 the q-arm. From these 44 q-arm clones, 77% (34/44) were included in the LM-PAM classifier (Table 2; marked bold-italic). The rest of the BAC clones (50/84) in the LM-PAM classifier were distributed over 20 different chromosomal locations (Figure 2; lower panel and (Additional file 2 table S1).

**Table 2:** BAC locations on the CGH array representative for chromosome 20

BAC-clone	Chr	Arm	BAC-clone	Chr	Arm	BAC-clone	Chr	Arm
GS-82-O2	20	p	***RP11-348I14***	***20***	***q***	***RP11-347D21***	***20***	***q***
GS-1061-L1	20	p	RP3-324O17	20	q	***RP1-155G6***	***20***	***q***
RP5-852M4	20	p	RP5-857M17	20	q	***RP3-470L14***	***20***	***q***
RP11-314N13	20	p	RP1-310O13	20	q	***RP4-791K14***	***20***	***q***
RP4-686C3	20	p	RP11-410N8	20	q	RP5-1185N5	20	q
RP4-741H3	20	p	***RP5-1085F17***	***20***	***q***	RP4-530I15	20	q
RP4-599I11	20	p	RP4-733O23	20	q	***RP5-994O24***	***20***	***q***
RP4-764O22	20	p	***RP5-1137F22***	***20***	***q***	***RP4-715N11***	***20***	***q***
RP5-1140M3	20	p	***RP11-353C18***	***20***	***q***	RP4-724E16	20	q
RP4-811H13	20	p	***RP11-234K24***	***20***	***q***	***RP5-1075G21***	***20***	***q***
RP11-204H22	20	p	***RP3-469A13***	***20***	***q***	***RP5-1162C3***	***20***	***q***
RP4-742J24	20	p	***RP4-633O20***	***20***	***q***	***RP11-6L15***	***20***	***q***
RP11-104O6	20	p	***RP11-122O1***	***20***	***q***	***RP5-1167H4***	***20***	***q***
RP11-526K24	20	p	***RP4-600E6***	***20***	***q***	RP5-1153D9	20	q
RP5-822J19	20	p	***RP5-892M9***	***20***	***q***	***RP11-46O3***	***20***	***q***
RP3-348M17	20	p	***RP4-796I11***	***20***	***q***	***RP13-379L11***	***20***	***q***
RP11-504H3	20	p	***RP1-128O17***	***20***	***q***	***RP1-309F20***	***20***	***q***
RP1-167O22	20	p	***RP1-232N11***	***20***	***q***	***RP5-827E24***	***20***	***q***
RP4-788L20	20	p	***RP1-138B7***	***20***	***q***	***RP5-1107C24***	***20***	***q***
RP1-234M6	20	p	***RP5-1028D15***	***20***	***q***	***RP4-563E14***	***20***	***q***
RP5-1025A1	20	p	***RP3-337O18***	***20***	***q***	***RP13-152O15***	***20***	***q***
			RP5-1005L2	20	q	***GS-81-F12***	***20***	***q***

Bold-Italic: 20q probes used in the LM-PAM classifier

BAC-clone = bacterial artificial chromosome -clone

Chr. = chromosome

The second classifier was trained to distinguish the PM group from the M0 group. A PM-PAM classifier was able to separate the PM group from the M0 with 90% accuracy (Additional file 3 figure S2, misclassification error 10%). This PM-PAM classifier included 140 BAC clones and was, unlike the LM-PAM classifier, employing probes spread over the whole genome (Additional file 4 table S2).

## Discussion

One of the challenges in CRC therapy lies in the early detection and treatment of CRC metastases. Elucidation of the molecular and cellular mechanisms of developing metastases will play an important role in future diagnostic and therapeutic interventions. A well established screening method to detect the genetic changes that underlie carcinogenesis is comparative genomic hybridization and was first introduced by Kallionemi and colleagues in 1992 [32]

In the present study we examine genome wide chromosomal aberrations in primary CRC to identify molecular markers predictive for liver metastases. The method we chose to examined genome wide chromosomal aberrations is a published and publically available method to look for recurrent copy number alterations and differential copy number alteration[31]. The KC-SMART method was applicable to our research questions and therefore used. An important principle in the analysis of this 20q amplification is that a threshold value can be (arbitrarily) set, but the logistic regression shows a continuous relationship between the aCGH measurement and the risk of a liver metastasis. Binarizing the predictive variable would cause information loss in this case. We believe that there is more information in the continuous mean log2 value of the 20q probes than just an on/off call.

To the best of our knowledge, this is the first study reporting a classifier predictive for liver metastases in primary CRC based on genomic aberrations. Our data demonstrate that primary colorectal tumors that developed liver metastases are characterized by an amplification of chromosome 20q. This amplification of chromosome 20q occurred significantly more often in the LM group compared to the other groups (PM and M0).

In this study there were significantly more left sided and rectum tumors in the LM group as compared to the PM group. So far, however, there is no evidence that peritoneal metastasis are related to the location of the primary colorectal tumor. These primary lesions are often characterized as mucinous T4 tumors which spread tumor cells into the peritoneal cavity[33]. It might be that left sided and rectum tumors could modify the penetrance of chromosome 20q and as result more frequently develop liver metastases. This could contribute to the fact that left sided and rectal tumors in our study result more frequently into liver metastases whereas right sided tumors more often into peritoneal metastases.

We created a classifier to predict liver metastases in patients with CRC. This LM-PAM classifier was able to identify patients who would develop liver metastases and resulted in an AUC of 0.86. Although the classifier was constructed by employing cross-validation to obtain unbiased error estimates, this result should be confirmed in an independent validation set. Logistic regression analysis on the mean 20q copy number ratio showed for every 0.1 log_2 _20q amplification an odds ratio of 16.2 (90% CI, 2.3-141.2) for developing liver metastases.

Amplification of chromosome 20q has been identified in several tumor types including breast, ovary, bladder, pancreas and stomach [32,34-38]. In colorectal cancer, chromosome 20q has been related to tumor progression, liver metastases and as an indicator of worse patient survival [10-13,16,29,39-42]. Knösel et al. created a progression model to identify relevant chromosomal imbalances specific for metastases but this model was not created to predict site of metastases in primary CRC [43]. Recently, Nakao et al. showed that specific copy number aberrations were linked to nodal metastases and reported a significant difference in 20q amplification in primary colorectal tumors between patients who had liver metastases at time of surgery and those who had no liver metastases. Unfortunately, they did not propose a prediction model for liver metastases [44,45]. Diep et al. presented a genetic pathway for CRC progression based on a meta-analysis of 31 CGH studies. They identified specific chromosomal alterations linked to different stages of tumor progression and liver metastases and found that the majority of chromosomal alterations were present in both primary carcinomas and liver metastases. They showed that the number of alterations increases in the transition from primary carcinomas to liver metastases. Furthermore, they showed that the role of chromosome 20q was evident in patients with Dukes D classification [9].

Hence, across all studies, chromosome 20q is of importance in the development of liver metastases. Therefore, more understanding of the candidate genes located on chromosome 20q may guide us to understand the biological mechanisms in the development of liver metastases. So far, several genes located on 20q have been described to play an important role in tumor progression and liver metastases. For example genes such as *CAS/CSE1L, NABC1, ZNF217, Aurora2 (BTAK, STK15), LIVIN, PTK6, HD54, EEF1A2, PSMA7, TPX2, AURKA *and the ubiquitin-conjugating enzyme *E2C (UBE2C) *[10-13,45-51].

These candidate genes located on chromosome 20q should be taken into account when examining new targeted therapeutic regimens for patients with CRC. For example, chromosome 20q amplification in CRC showed in an *in vitro *study response to Kinesin-5 Inhibitor. This inhibitor plays a role in the mitotic spindle function in the cell. Resistance to Kinesin was dominated by amplification of chromosome 20q. It was suggested that *AURKA *and the *TPX2 *gene located on 20q were the genes resistance for the Kinesin-5 Inhibitor[51]. Amplification of 20q could therefore be a potential target for novel antimitotic cancer therapies.

In summary, organ specific CRC metastases localization can be predicted by a LM-PAM classifier on the basis of specific genomic aberrations in the primary colorectal tumor. The validation of the LM-PAM classifier will further potentiate its role as a tool in clinical practice. As a result, patients at risk for developing liver metastases should be frequently screened with modern imaging tools and are most likely to benefit from additional chemotherapy.

We show the possibility for specific CGH profiles to predict CRC metastases target organ with 80% precision. This is the first tool to do this and as such may provide the information to guide individual treatment protocols. In daily clinical practice q-PCR or FISH probes could be used on FFPE tissue for detecting patients at risk based on a 20q amplification. We are now developing an MLPA analysis of chromosome 20q to identify specific regions involved in the development of liver metastasis.

## Conclusion

The ability to predict liver metastases based on specific genomic aberrations in the CRC is important for understanding the biology of the tumor, to perform tailored follow-up and eventually develop targeted therapy in patients with CRC. Further research should be done by investigation specific target regions of chromosome 20q.

## Competing interests

The authors declare that they have no competing interests.

## Authors' contributions

SCB: participated in the study design, coordination and data collection, carried out the CGH arrays, participated in the statistical analysis and drafted the manuscript. CK: performed the statistical analysis, participated in the study design and helped to draft the manuscript. G-JL: participated in the study design, participated in the tissue recruitment and participated in collecting patient data. LMB: helped to perform the CGH analysis. SAJ: participated in the statistical analysis and helped to perform the CGH analysis and helped to draft the manuscript. EHvB: participated in the statistical analysis and helped to perform the CGH analysis and helped to draft the manuscript. VJV: participated in the study design and helped to draft the manuscript. HM: participated in the tissue recruitment. LFW: helped to perform the statistical analysis participated in the study design and helped to draft the manuscript. M-LFvV: participated in the study design, performed the histological classification and helped to draft the manuscript. RAEMT: participated in the study design and helped to draft the manuscript. LJv 'tV: conceived of the study, participated in the study design, performed the supervision and coordination and helped to draft the manuscript. All authors read and approved the manuscript.

## Pre-publication history

The pre-publication history for this paper can be accessed here:

http://www.biomedcentral.com/1471-2407/10/662/prepub

## Supplementary Material

Additional file 1 **Figure S1**. Significant gains (FDR < 0,05) and losses specific for the LM group. Click here for file

Additional file 2 **Table S1**. BAC locations (84) used for the LM-PAM classifier (Figure 4). Click here for file

Additional file 3 **Figure S2**. PAM analysis of the Peritoneal Metastases (PM) (2) versus the No metastases (M0) group (3). Arrow: location of the classifier and number (140) of BAC clones in classifier. Click here for file

Additional file 4 **Table S2**. BAC locations (140) used for the PM-PAM classifier (additional figure S2). Click here for file
