# Supplementary figures and images for: Specific genomic aberrations in primary colorectal cancer are associated with liver metastases
Source: BMC Cancer. 2010 Dec 2;10:662. doi: 10.1186/1471-2407-10-662 (PMC3027605; doi:10.1186/1471-2407-10-662)

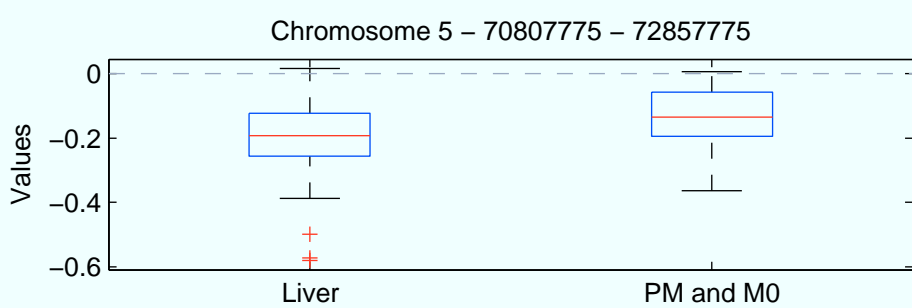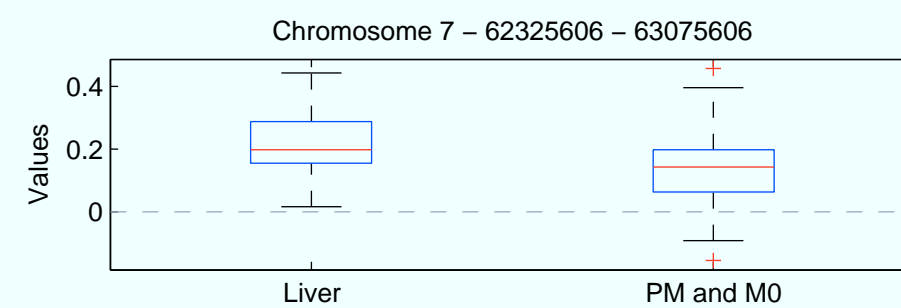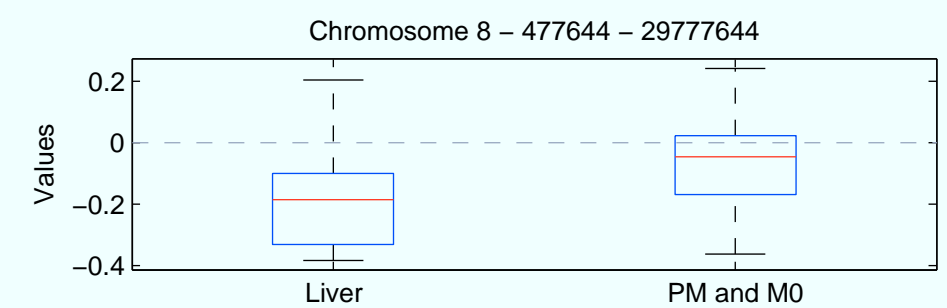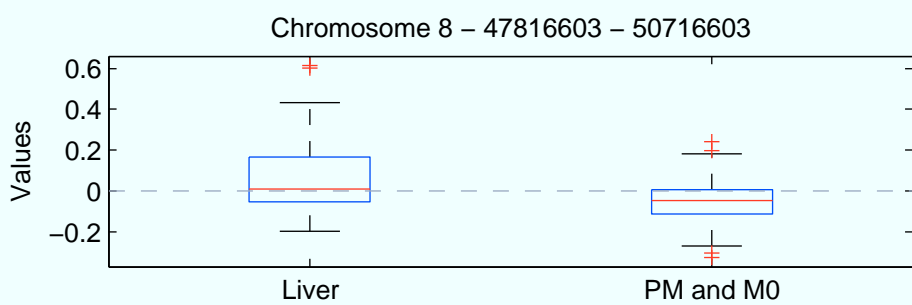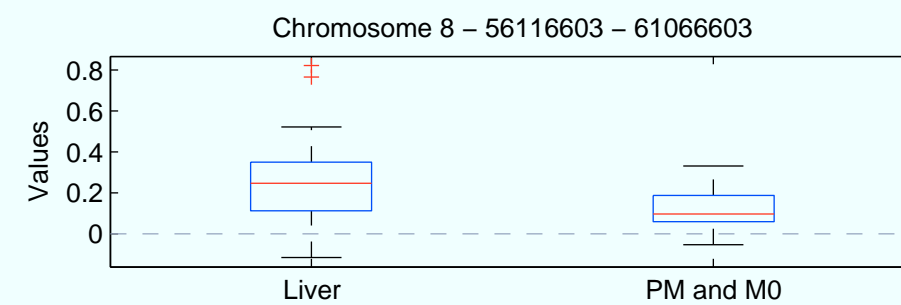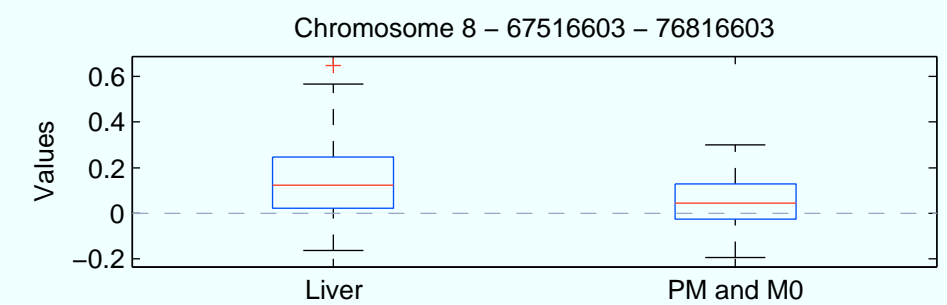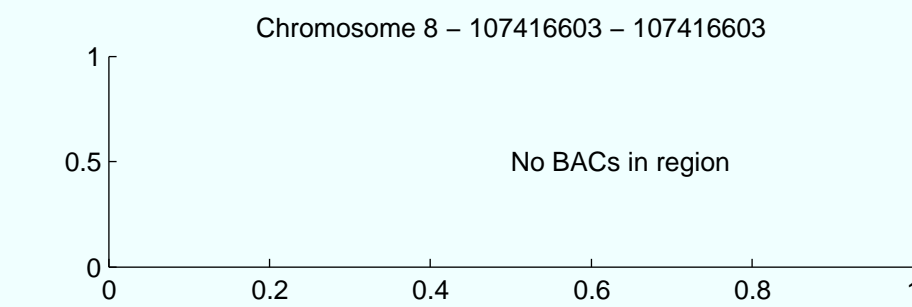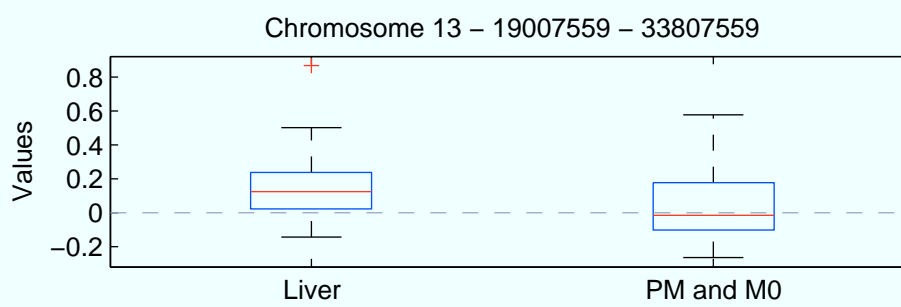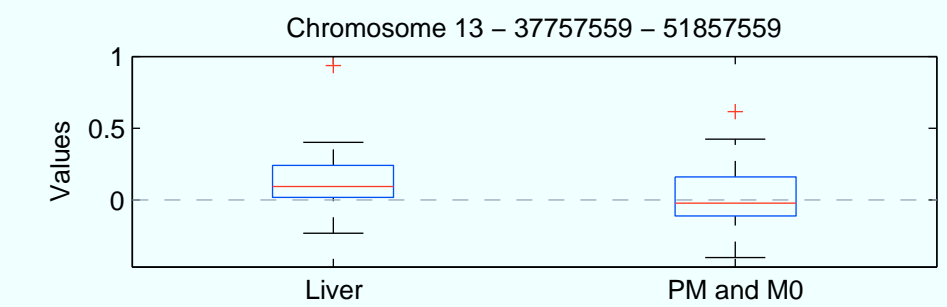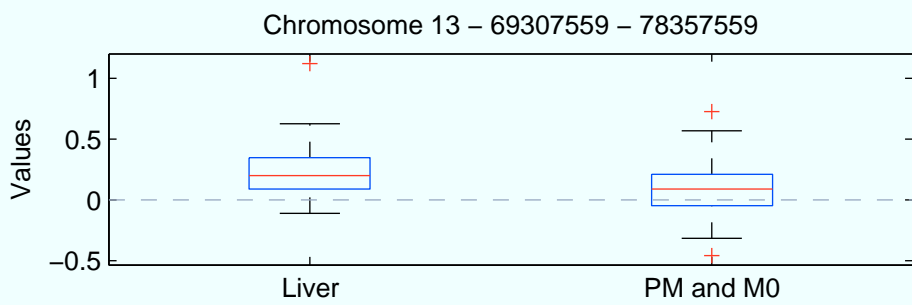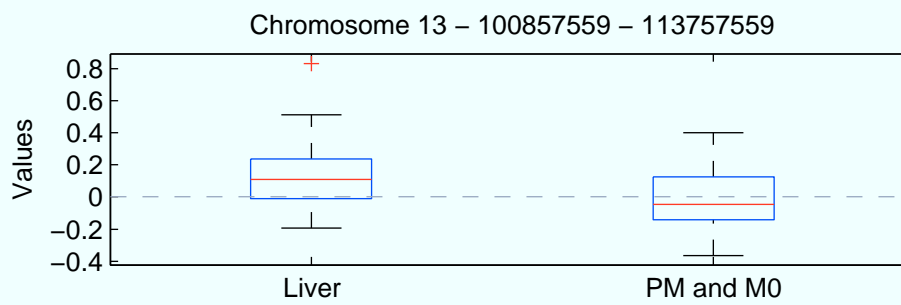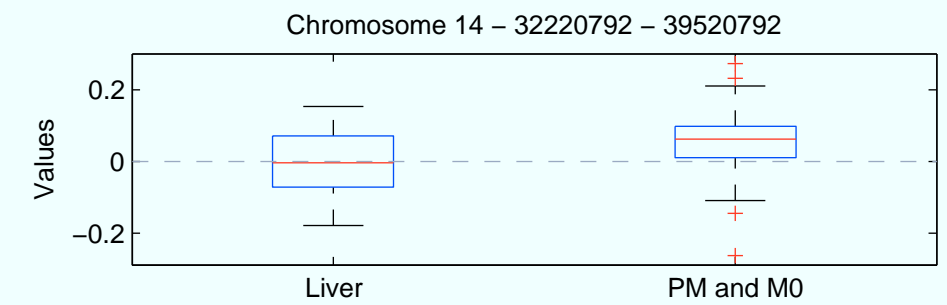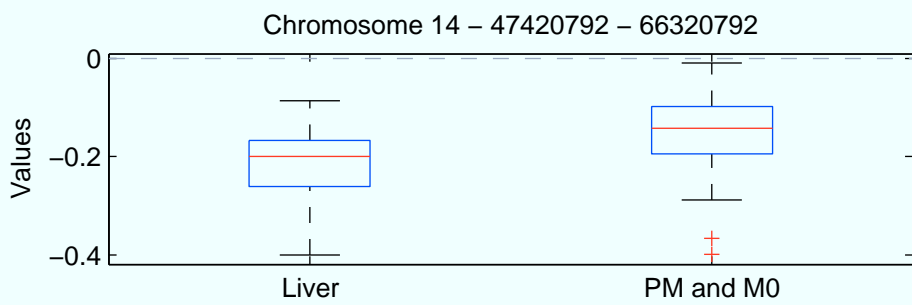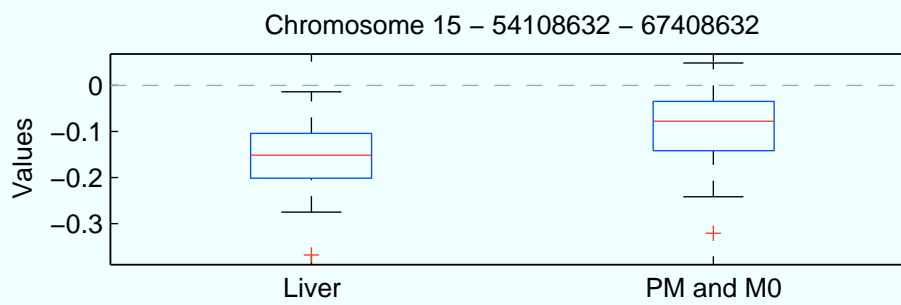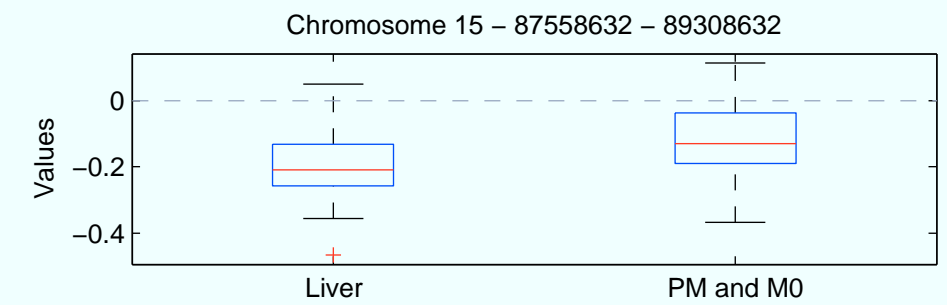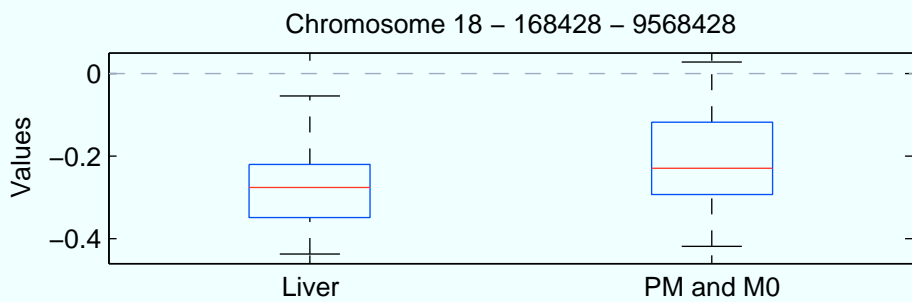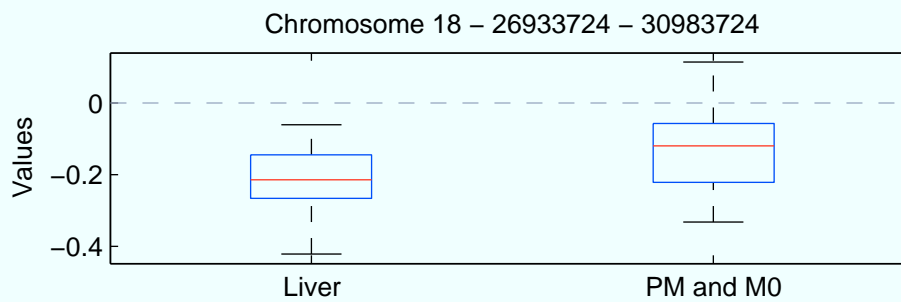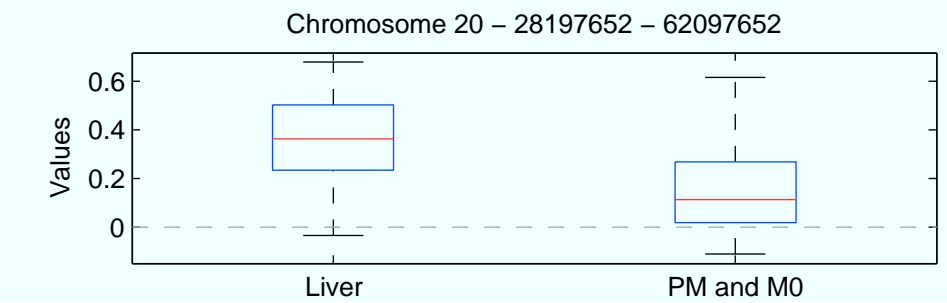

Supplement: Additional file 1 — Figure S1. Significant gains (FDR < 0,05) and losses specific for the LM group. [file 1471-2407-10-662-S1.PDF]

# PM-M0 Classifier

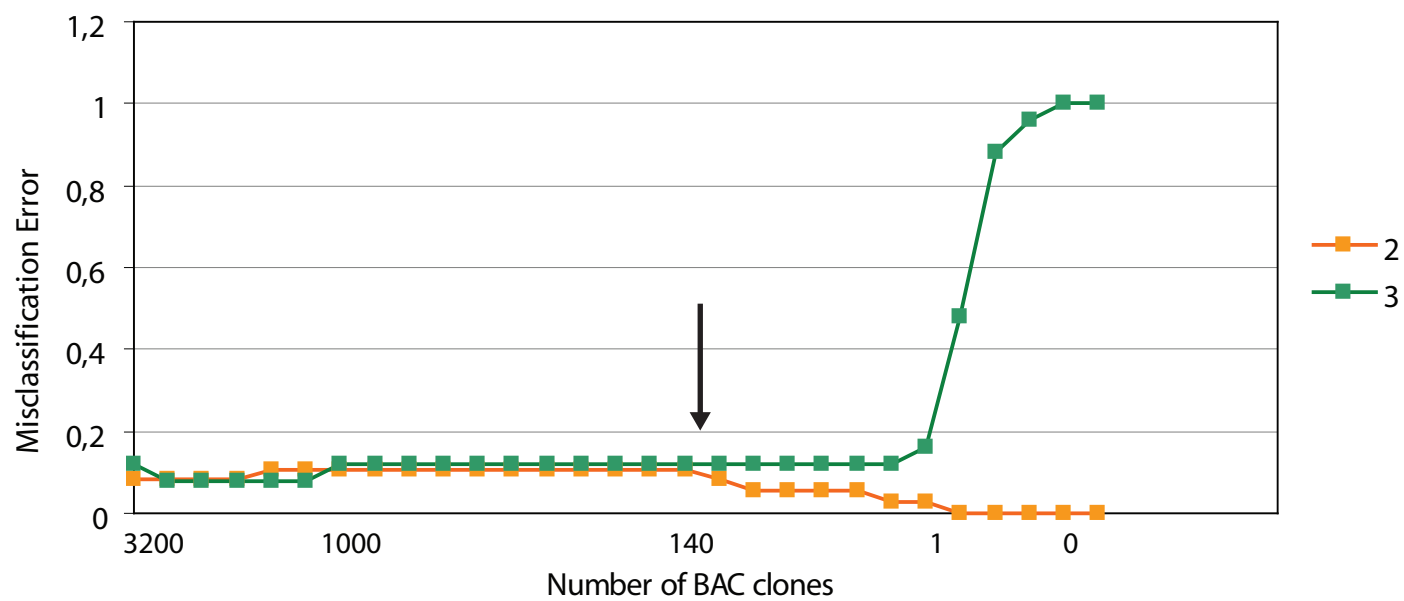

Supplement: Additional file 3 — Figure S2. PAM analysis of the Peritoneal Metastases (PM) (2) versus the No metastases (M0) group (3). Arrow: location of the classifier and number (140) of BAC clones in classifier. [file 1471-2407-10-662-S3.PDF]
