# Supplementary material for: Specific genomic aberrations in primary colorectal cancer are associated with liver metastases
Source: BMC Cancer. 2010 Dec 2;10:662. doi: 10.1186/1471-2407-10-662 (PMC3027605; doi:10.1186/1471-2407-10-662)
Supplement: Additional file 2 — Table S1. BAC locations (84) used for the LM-PAM classifier (Figure 4). [file 1471-2407-10-662-S2.PDF]

**Table S1: BAC locations (84) used for the LM-PAM classifier (Figure 5)**

| Location BAC clones | Location BAC clones | Location BAC clones |
|---|---|---|
| 102_RP11-62E9_chr8_1473555051 | 2059_RP11-200O17_chr3_636150301 | 2951_RP11-9B17_chr19_2687777845 |
| 193_RP1-138B7_chr20_2750287872 | 2067_RP11-202L1_chr8_1468869506 | 3015_RP11-376O14_chr7_1352514213 |
| 209_RP4-563E14_chr20_2769688074 | 2068_RP11-211C9_chr8_1400146043 | 3026_RP11-365N19_chr14_2296821432 |
| 213_RP4-600E6_chr20_2746277071 | 2071_RP11-203D9_chr14_2278350115 | 3045_RP5-1028D15_chr20_2750397993 |
| 218_RP11-562H1_chr18_2598173021 | 2079_RP11-204J18_chr3_552469821 | 3060_RP1-155G6_chr20_2755750726 |
| 219_RP11-575G13_chr12_2047379711 | 2080_RP11-213G21_chr4_727925587 | 3260_RP3-441A12_chr6_1099783083 |
| 279_GS-196-F4_chr3_688634672 | 2093_RP11-382A21_chr3_500267730 | 3402_RP11-338H14_chr11_1908370894 |
| 346_RP5-1085F17_chr20_2739512064 | 2111_RP11-419J16_chr18_2578900010 | 3451_RP11-60K23_chr14_2248072210 |
| 350_RP5-1162C3_chr20_2760939310 | 2353_RP1-128O17_chr20_2748649128 | 3452_RP11-114M5_chr8_1451091266 |
| 394_RP11-408L13_chr13_2127325623 | 2357_RP1-232N11_chr20_2749397642 | 3584_RP11-6L15_chr20_2761194811 |
| 454_RP11-388N6_chr9_1649052795 | 2358_RP5-827E24_chr20_2768143915 | 3592_RP11-234K24_chr20_2742931132 |
| 647_RP11-74H8_chr17_2552145029 | 2361_RP1-309F20_chr20_2765550773 | 3596_RP11-347D21_chr20_2754693875 |
| 738_RP5-1137F22_chr20_2740289780 | 2365_RP3-337O18_chr20_2752593744 | 3600_RP11-353C18_chr20_2742445620 |
| 750_RP5-892M9_chr20_2746923711 | 2366_RP5-994O24_chr20_2758208532 | 3739_RP11-416K7_chr17_2532916751 |
| 753_RP4-796I11_chr20_2748013080 | 2369_RP3-469A13_chr20_2743485675 | 3743_GS-820-M16_chr14_2299774206 |
| 893_RP5-1167H4_chr20_2763065357 | 2440_RP11-120E20_chr11_1817143803 | 3747_RP11-418P19_chr5_981786862 |
| 953_RP11-406G20_chr13_2185652709 | 2509_RP3-470L14_chr20_2755847844 | 3750_RP11-447J13_chr3_576380372 |
| 954_RP11-564N10_chr13_2181692810 | 2602_RP11-345K9_chr9_1627777771 | 3956_RP11-24N10_chr15_2361817189 |
| 1034_RP5-991O23_chr8_1396947689 | 2758_RP11-413E1_chr1_89928890 | 4054_RP11-545G16_chr11_1946016857 |
| 1238_RP11-336N16_chr8_1394538773 | 2760_RP11-293B7_chr1_181934217 | 4078_GS-1061-L1_chr20_2708841122 |
| 1373_RP11-533H15_chr11_1905460642 | 2765_RP11-8J9_chr1_46963251 | 4103_RP1-144M5_chr8_1426127400 |
| 1424_RP11-46O3_chr20_2763512110 | 2837_CTB-187L3_chr19_2660341767 | 4128_RP13-379L11_chr20_2764678884 |
| 1428_RP11-122O1_chr20_2745453461 | 2893_RP4-633O20_chr20_2744543987 | 4132_RP13-152O15_chr20_2770797112 |
| 1435_RP11-509D8_chr9_1542830142 | 2894_RP5-1075G21_chr20_2760848633 | 4172_RP11-257P13_chr14_2284425019 |
| 1436_RP11-348I14_chr20_2736893664 | 2895_RP11-47L18_chr22_2838396413 | |
| 1505_RP11-99A1_chr18_2619364871 | 2897_RP4-715N11_chr20_2759434108 | |
| 1691_RP11-567J20_chr8_1441162347 | 2898_RP5-1107C24_chr20_2768616701 | |
| 1918_GS-81-F12_chr20_2771047036 | 2913_RP4-791K14_chr20_2756121696 | |
| 2045_RP11-102G17_chr7_1369420200 | 2925_RP11-559M6_chr14_2256416757 | |
| 2055_RP11-90J19_chr15_2346013719 | 2937_RP11-561B11_chr14_2229352670 | |
