# Supplementary material for: Specific genomic aberrations in primary colorectal cancer are associated with liver metastases
Source: BMC Cancer. 2010 Dec 2;10:662. doi: 10.1186/1471-2407-10-662 (PMC3027605; doi:10.1186/1471-2407-10-662)
Supplement: Additional file 4 — Table S2. BAC locations (140) used for the PM-PAM classifier (additional figure S2). [file 1471-2407-10-662-S4.PDF]

Table S2: BAC locations (140) used for the PM-PAM classifier (Figure S2)

Location BAC clones	Location BAC clones	Location BAC clones	Location BAC clones
1530_RP11-228F17_chr2_403798781	1154_RP11-158L8_chr2_421313654	630_RP11-122d19_chr3_544298787	2063_RP11-202H2_chr12_2033535737
171_RP11-207K8_chr10_1708032151	3579_RP1-85M6_chr11_1846526891	2760_RP11-293B7_chr1_181934217	543_RP11-490O6_chr16_2412846001
3072_RP11-35A5_chr8_1453475347	4161_CTC-1554D6_chr5_993134156	1236_RP11-359B12_chr12_1948866061	1576_RP11-423P14_chr4_755569863
567_RP11-317C13_chr7_1386349112	1694_RP11-164M8_chr18_2606155320	2798_RP11-64O13_chr3_643449215	3863_RP11-172B9_chr2_445059886
2708_RP11-502N13_chr12_1962515395	1963_RP11-545E17_chr9_1668414713	3231_RP11-290L7_chr9_1576910673	2940_RP11-410P24_chr21_2804031555
996_GS-112-N13_chr9_1673420763	652_RP11-84C16_chr19_2694867205	2342_RP11-129O7_chr10_1703309401	3692_RP11-206L19_chr11_1825232827
1774_RP11-342K2_chr17_2547403452	1471_RP11-239A17_chr12_1964777861	4312_RP11-57I6_chr5_904695482	1067_RP11-268E1_chr9_1583756056
4228_RP11-210N13_chr12_1990402645	1768_RP11-361M10_chr15_2371592122	4035_RP11-311L3_chr2_399155884	2609_RP11-176L21_chr9_1628539320
2294_CTC-325L16_chr19_2682493645	2167_RP11-485A5_chr11_1936835064	1000_RP11-165P4_chr9_1660786097	1284_RP11-105D1_chr15_2349805070
3251_RP4-726A1_chr6_1186276823	3541_GS-8-L3_chr2_247579770	1673_RP11-430E17_chr18_2592800248	1829_RP11-16A6_chr5_912391694
2707_RP11-491D6_chr3_527287204	4313_RP11-168K7_chr2_248947834	251_RP11-7M4_chr5_918058561	8_RP11-497G19_chr12_2063493107
2712_RP11-502P9_chr7_1244594154	2725_RP11-48O9_chr11_1846443031	1268_RP11-24H13_chr4_724272753	2834_CTC-444N24_chr19_2707320161
980_RP11-123J20_chr9_1555803791	2688_RP11-475I24_chr9_1582176931	16_RP11-500K7_chr3_639409303	3653_RP11-266L10_chr2_402806421
3152_RP11-143H20_chr9_1670761853	3500_RP11-316G9_chr2_336916872	2673_RP11-80p20_chr3_629823395	123_CTC-482H14_chr19_2649926190
4223_RP11-201J10_chr2_304466213	2432_RP11-118O11_chr3_560968138	2021_RP11-268O21_chr19_2647868310	2141_RP11-144K9_chr17_2496851833
1251_RP11-21B21_chr16_2488303750	984_RP11-132E11_chr9_1554896623	1250_RP11-17C4_chr4_848341450	962_RP11-49M9_chr11_1913158426
2730_RP11-164G17_chr14_2264956562	4270_RP11-451C2_chr2_349257291	4176_RP11-258N2_chr4_771293315	4225_RP11-220C23_chr11_1854480803
3687_RP11-195A20_chr5_980008537	815_RP11-115H23_chr2_445524822	1056_RP11-757F24_chr22_2839246140	1258_RP11-17L5_chr15_2378112155
2971_RP11-115D19_chr4_780541610	3219_RP11-211N8_chr9_1584530710	4149_CTC-1277H1_chr11_1831038483	451_RP11-20P5_chr9_1565952178
1748_RP11-25F7_chr11_1889525542	3691_RP11-196E5_chr17_2561112623	1828_RP11-137P5_chr5_886569311	1339_RP11-307P22_chr14_2254740821
4034_RP11-301K6_chr2_405497003	4188_RP11-261A24_chr2_310539239	2282_CTC-251H24_chr19_2663156668	3514_RP11-540E4_chr8_1399659514
2286_CTC-263A14_chr5_1035393759	576_RP11-472K18_chr8_1535813994	2617_RP11-205K6_chr9_1666161830	2764_RP11-108M21_chr1_185528713
2727_RP11-314A5_chr3_542060429	2097_RP11-397E9_chr21_2815856872	1185_RP11-30G4_chr3_519090926	4171_RP11-243M7_chr11_1823144524
2174_RP11-478P5_chr17_2559812620	2847_RP5-831G13_chr1_109850591	2065_RP11-220N20_chr17_2532205208	4203_RP11-89F17_chr3_541675764
2951_RP11-9B17_chr19_2687777845	3487_RP11-304A10_chr7_1241728959	14_RP11-479F13_chr2_308582345	4232_RP11-211G17_chr4_756780324
2344_RP11-348J12_chr10_1772919307	3215_RP11-195F19_chr9_1572557550	1051_RP11-185P18_chr14_2289868396	423_RP11-26H10_chr3_570416301
2340_RP11-345K20_chr10_1752960097	3683_RP11-193I17_chr7_1363996677	4261_RP11-411B10_chr18_2582802941	1340_RP11-317A20_chr12_2077012779
3216_RP11-744K17_chr17_2511827200	3402_RP11-338H14_chr11_1908370894	783_RP11-9A1_chr3_554479095	2431_RP11-111P21_chr3_535668956
1921_RG-41-L13_chr9_1538429874	2011_RP11-244E6_chr2_418420755	3867_RP11-173C1_chr2_286347209	3755_RP11-3J1_chr4_708500539
799_RP11-10F11_chr7_1289454873	2865_RP4-783C10_chr1_37965791	938_RP11-521L15_chr13_2147735035	3503_RP11-308J14_chr18_2577616814
2731_RP11-320N7_chr12_1952024003	2675_RP11-15E1_chr9_1580779843	122_CTC-260F20_chr19_2664341674	3761_RP11-91K8_chr3_624896856
3686_RP11-227F19_chr4_731135293	2071_RP11-203D9_chr14_2278350115	1709_RP11-156L14_chr17_2548150579	254_RP11-3J21_chr8_1484789412
1525_RP11-215A20_chr18_2627396707	2713_RP11-469N6_chr11_1947544202	4264_RP11-356B18_chr15_2387347368	4307_RP11-21B14_chr11_1873080916
3280_RP11-266C7_chr6_1220193965	1955_RP1-236L2_chr5_1010560563	1609_RP11-121L11_chr5_884487788	
512_RP11-395E19_chr9_1578539983	2139_RP11-20F20_chr1_57542691	3415_RP11-21C5_chr8_1461089125	
